# Regional and Gender Differences in Years with and without Mobility Limitation in the Older Population of Thailand

**DOI:** 10.1371/journal.pone.0153763

**Published:** 2016-05-03

**Authors:** Benjawan Apinonkul, Kusol Soonthorndhada, Patama Vapattanawong, Carol Jagger, Wichai Aekplakorn

**Affiliations:** 1 Faculty of Physical Therapy, Mahidol University, Salaya, Phutthamonthon, Nakhon Pathom, Thailand; 2 Institute for Population and Social Research, Mahidol University, Salaya, Phutthamonthon, Nakhon Pathom, Thailand; 3 Newcastle University Institute for Ageing and Institute of Health and Society, Newcastle University, Newcastle upon Tyne, United Kingdom; 4 Department of Community Medicine, Faculty of Medicine, Ramathibodi Hospital, Mahidol University, Bangkok, Thailand; National Institute for Viral Disease Control and Prevention, CDC, China, CHINA

## Abstract

**Objectives:**

To examine gender and regional differences in health expectancies based on the measure of mobility.

**Methods:**

Health expectancies by gender and region were computed by Sullivan’s method from the fourth Thai National Health Examination Survey (2009). A total of 9,210 older persons aged 60 years and older were included. Mobility limitation was defined as self-reporting of ability to perform only with assistances/aids at least one of: walking at least 400 metres; or going up or down a flight of 10 stairs. Severe limitation was defined as complete inability to do at least one of these two functions, even with assistances or aids.

**Results:**

At age 60, females compared to males, spent significantly fewer years without mobility limitation (male-female = 3.2 years) and more years with any limitation (female-male = 6.7 years) and with severe limitation (female-male = 3.2 years). For both genders, years lived with severe limitation were remarkably constant across age. Significant regional inequalities in years lived without and with limitation were evident, with a consistent pattern by gender in years free of mobility limitation (Central ranked the best and the North East ranked the worst). Finally, both males and females in the South had the longest life expectancy and the most years of life with severe mobility limitation.

**Conclusion:**

This study identifies inequalities in years without and with mobility limitations with important policy implication.

## Introduction

The world’s population is ageing and Thailand is no exception. Life expectancy at birth for the Thai population has seen a continuous rise, from 60 years in 1967 [1] to 72 years for males and 78 years for females in 2015 [2] and with an increasing proportion of older people, from five percent of the total population in 1970 [3] to 16 percent by 2015 [2]. With population ageing, non-communicable and degenerative diseases have steadily increased around the world, including Thailand, resulting in long-term health consequences creating a need for long-term treatment and care [4].

The key concern accompanying increases in life expectancy is whether the additional years are spent in good health. According to the International Classification of Functioning, Disability and Health model (ICF model) [5], health/ill-health is not only associated with the absence/presence of diseases but is also related to the concepts of functioning (referring to all body functions), activities, and participation. Moreover the World Health Organization’s conceptual framework for healthy ageing has functional ability and intrinsic capacity at the core, with functional ability being the interaction between intrinsic capacity and the environment [6].

Mobility is one of the most basic functions for independent living in old age. Mobility limitation is common in older adults, with multifactorial causes, i.e. declining physical activity, obesity, gait and balance impairment, and having chronic diseases, such as diabetes and arthritis [7]. Mobility limitation adversely affects physical, psychological, and social aspects of older adults’ life and is more strongly correlated with health-related quality of life than a medical comorbidity index [8]. In addition, there are significant associations between mobility limitation, quality of life, and depressive symptoms [8,9] and mobility limitation leads to diminished social participation resulting in isolation and loneliness [10]. Loss of independent mobility is associated with higher rates of functional disability, placement in nursing home, and mortality in older adults [11]. Despite this, most studies on the epidemiology of ageing have focused on measures of disease status (both physical and mental), and disability in Activities of Daily Living (ADL) and Instrumental Activities of Daily Living (IADL) [12]. There is an increasing need for knowledge and evidence-based policy to promote mobility in the older adults to ensure well-being and quality of life.

Health expectancy is an index of population health which extends measures of life expectancy to account for quality of life lived [13,14]. Health expectancy combines information on mortality with epidemiologic data to estimate length of time spent in different states of health until death [14, 15]. In Thailand, health expectancies have been computed based on chronic diseases [16], cognitive impairment [16], depression [16], ADL and IADL disability [16–19], as well as different states of perceived health [20] at older ages with results varying by the measure used and the time period of the study. All studies have focused on gender differences and/or trends over time. Nevertheless, estimates of health expectancy based on measure of mobility, are still unknown, particularly for the Thai population. This study therefore uses the most recent Thai national data from 2009 to estimate years of life at older ages in different states of mobility limitation, and examines gender and regional differences in these health expectancies.

## Materials and Methods

### Data

Data on mobility performances were obtained from the fourth Thai National Health Examination Survey (NHES IV) in 2009 conducted by the Health Systems Research Institute. This survey is a cross-sectional design using stratified four-stage sampling to provide nationally and regionally representative samples of the Thai population. The sampling method has been described elsewhere [21] but briefly the sampling units in each stage included: i) five provinces in each of the four regions; ii) three to five districts for each selected province and Bangkok; iii) 13–14 electoral units in municipality areas or villages in non-municipal areas for each selected district; iv) individuals aged 15 years and older by selected electoral units and villages, gender, and age group. A total of 20,450 samples were obtained but the present study focused on 9,210 older persons aged 60 years and over (with a 95% response rate). For data collection, various methods were used, including a face-to-face interview, functional tests, physical examinations, and laboratory tests with data quality assured. The NHES IV was approved by the Ethical Review Committee for Research in Human Subjects, Ministry of Public Health. And, all participants provided written informed consent.

To compute life expectancy, we took the number of deaths from the Thai vital registration system, the most significant source of mortality data since civil registration law mandates every birth and death event to be registered at the district offices or municipality registrars. Population denominators by region were obtained from the civil registration system reported by Department of Provincial Administration (DOPA).

### Health measures

Mobility performance was measured by two functions; (i) walking at least 400 metres without resting, and (ii) going up or down a flight of 10 stairs without resting. Participants were defined as having mobility limitation (any severity) if they reported inability (complete or partial) to do at least one of these two functions without human assistances or technical aids. Severe mobility limitation was defined as complete inability to do at least one of these two functions, even with assistances or aids.

### Statistical methods

Health expectancies were calculated by the Sullivan method [22,23] as previously described. The calculation method has been described elsewhere [16]. Briefly, the period life tables by gender and region were calculated from the age- and sex-specific death rates. This required some adjustment in (i) the number of deaths (for unknown age of death and under-registration) and (ii) the age- and sex-specific death rates for the very old. The age- and sex-specific prevalence of mobility limitation for the country as a whole and each region were then applied to divide the number of person years lived in the given age interval (from the period life table) into years lived with and without mobility restriction. We calculated the variance and used z-statistics to test differences in health expectancies following established methods [22].

## Results

### Characteristics

Females were a slight majority of participants aged 60 years and older in the NHES IV, accounting for 51 percent of participants aged 60 and older. The mean age of participants was 69.4 years (SD = 6.9) for males and 69.6 years (SD = 7.1) for females, respectively.

### Prevalence of mobility limitation

The age- and sex- specific prevalence of mobility limitation by regions for the Thai population aged 60 years and over in 2009 is shown in Table 1 (for any mobility limitation) and Table 2 (for severe limitation). The prevalence of mobility limitation increased with age in both genders but females had higher prevalence of mobility limitation than males of the same age. Most noticeable is that the prevalence in females was approximately twice that in males up to age 80 years. Although differences in the prevalence of mobility limitation were evident between the regions, patterns were not consistent across age.

**Table 1:** Age- and sex- specific prevalence*(in percent) of mobility limitation for Thailand, and the 4 regions and Bangkok, 2009 (95%CIs in parentheses).

Age (Years)	60–64	65–69	70–74	75–79	80+
Country					
Males^a^	10.5 (8.8–12.1)	14.6 (12.1–17.2)	21.0 (23.9–35.3)	29.6 (23.9–35.3)	53.3 (46.8–59.9)
Females^b^	30.4 (27.5–33.3)	35.4 (32.0–38.9)	48.8 (45.1–52.5)	55.1 (50.5–59.8)	77.4 (73.1–81.8)
Bangkok					
Males	4.7 (1.6–7.8)	6.4 (1.8–11.1)	14.4 (5.8–22.9)	16.4 (2.8–30.0)	51.7 (38.1–65.3)
Females	19.7 (15.7–23.7)	27.8 (22.5–33.1)	45.4 (37.6–53.2)	37.9 (30.8–45.1)	76.4 (61.9–91.0)
Central					
Males	4.7 (2.4–7.0)	11.2 (7.3–15.0)	12.8 (9.5–16.0)	18.7 (9.6–27.9)	40.9 (32.4–49.4)
Females	26.9 (21.1–32.6)	23.9 (18.5–29.2)	39.3 (30.7–47.9)	43.8 (31.7–55.8)	67.6 (60.2–75.0)
North					
Males	12.9 (8.7–17.1)	13.3 (8.9–17.8)	19.2 (12.9–25.5)	39.2 (33.1–45.4)	54.2 (42.1–66.2)
Females	33.4 (29.1–37.7)	33.6 (27.4–39.9)	45.9 (38.1–53.6)	58.7 (53.9–63.4)	77.7 (69.1–86.4)
North East					
Males	13.1 (10.5–15.7)	19.1 (15.3–22.9)	32.9 (25.4–40.4)	36.8 (27.6–45.9)	66.6 (51.2–81.9)
Females	35.3 (31.1–39.6)	49.9 (44.9–54.8)	57.1 (52.6–61.6)	60.0 (53.1–66.8)	86.9 (80.6–93.2)
South					
Males	15.7 (10.6–20.7)	21.7 (14.2–29.1)	19.0 (15.1–22.8)	37.8 (28.9–46.6)	36.6 (26.6–46.5)
Females	27.6 (22.0–33.2)	30.5 (24.2–36.7)	52.3 (43.9–60.8)	70.0 (60.1–79.8)	74.0 (63.3–84.8)

*With sampling weighted

^a^Sample size: Age 60–64 = 1,348, Age 65–69 = 1,150, Age 70–74 = 938, Age 75–79 = 628, Age 80+ = 442

^b^Sample size: Age 60–64 = 1,428, Age 65–69 = 1,131, Age 70–74 = 969, Age 75–79 = 683, Age 80+ = 493

Source: Author’s computations from the NHES IV

**Table 2:** Age- and sex- specific prevalence*(in percent) of severe mobility limitation for Thailand, and the 4 regions and Bangkok, 2009 (95%CIs in parentheses).

Age (Years)	60–64	65–69	70–74	75–79	80+
Country					
Males	3.3 (2.4–4.3)	4.7 (3.2–6.1)	5.7 (4.1–7.3)	10.3 (7.4–13.2)	24.7 (19.5–29.9)
Females	9.4 (7.8–11.0)	12.0 (9.9–14.2)	16.4 (13.9–18.9)	26.0 (21.9–30.1)	41.7 (37.3–46.1)
Bangkok					
Males	2.2 (0.3–4.1)	1.5 (0.4–2.7)	4.2 (1.2–7.2)	8.0 (1.3–14.7)	23.8 (11.6–35.9)
Females	12.2 (8.4–16.0)	9.2 (5.8–12.7)	9.5 (4.7–14.3)	23.0 (14.3–31.7)	65.4 (52.1–78.7)
Central					
Males	2.0 (0.6–3.5)	3.6 (1.2–6.1)	3.6 (0.9–6.2)	6.0 (1.8–10.2)	7.6 (2.3–12.9)
Females	7.8 (5.0–10.7)	11.7 (6.8–16.6)	16.9 (12.7–21.1)	19.0 (9.6–28.3)	34.3 (29.4–39.2)
North					
Males	2.2 (0.8–3.9)	6.8 (3.3–10.3)	5.9 (2.5–9.2)	12.6 (8.5–16.7)	33.5 (26.6–40.5)
Females	12.4 (9.0–15.8)	14.0 (9.0–19.0)	18.4 (12.8–24.0)	28.0 (21.6–34.3)	50.6 (41.0–60.1)
North East					
Males	3.8 (2.3–5.4)	4.2 (1.4–7.0)	8.1 (4.2–11.9)	12.8 (5.5–20.1)	28.8 (17.1–40.5)
Females	8.3 (5.5–11.1)	13.0 (9.4–16.5)	16.6 (12.1–21.2)	24.2 (16.7–31.6)	35.4 (28.2–42.7)
South					
Males	7.8 (3.9–11.8)	8.8 (4.4–13.1)	5.8 (3.1–8.4)	12.3 (7.5–17.1)	22.9 (15.4–30.4)
Females	7.9 (3.6–12.1)	9.5 (6.2–12.8)	18.0 (12.6–23.4)	42.7 (33.6–51.7)	42.5 (36.4–48.6)

*With sampling weighted

Source: Author’s computations from the NHES IV

### Life and health expectancy

At age 60, males and females could expect to live on average a further 19.1 and 22.6 year, respectively (Table 3). Females live longer than males at every age with the differences between females and males ranging from 3.5 years at age 60 to 1.5 years at age 80.

**Table 3:** Life expectancy and number of years lived in different states of mobility limitation at age 60, 65, 70, 75, and 80 by genders in Thailand, 2009.

	Males	Females
Age 60		
Life expectancy	19.13	22.58
Years lived		
Free of mobility limitation	14.40* (14.14–14.65)	11.18 (10.85–11.51)
With mobility limitation (any severity)	4.73 (4.48–4.98)	11.40* (11.07–11.73)
With severe mobility limitation	1.79 (1.60–1.98)	4.96* (4.65–5.27)
Age 65		
Life expectancy	15.86	18.78
Years lived		
Free of mobility limitation	11.18* (10.92–11.45)	8.26 (7.94–8.59)
With mobility limitation (any severity)	4.67 (4.41–4.94)	10.52* (10.19–10.84)
With severe mobility limitation	1.80 (1.60–2.01)	4.78* (4.45–5.10)
Age 70		
Life expectancy	12.92	15.31
Years lived		
Free of mobility limitation	8.31*(8.02–8.59)	5.66 (5.33–5.98)
With mobility limitation (any severity)	4.61 (4.32–4.90)	9.65* (9.33–9.98)
With severe mobility limitation	1.84 (1.61–2.06)	4.59* (4.26–4.94)
Age 75		
Life expectancy	10.47	12.32
Years lived		
Free of mobility limitation	5.90* (5.58–6.23)	3.78 (3.44–4.11)
With mobility limitation (any severity)	4.56 (4.24–4.89)	8.54* (8.21–8.87)
With severe mobility limitation	1.96 (1.70–2.24)	4.44* (4.06–4.81)
Age 80		
Life expectancy	8.6	10.08
Years lived		
Free of mobility limitation	4.01* (3.61–4.34)	2.28 (1.91–2.65)
With mobility limitation (any severity)	4.59 (4.19–4.99)	7.80* (7.43–8.18)
With severe mobility limitation	2.13 (1.78–2.47)	4.20* (3.77–4.64)

*Significantly higher than the other gender (p<0.001)

Despite female life expectancy being longer, males could expect on average 14.4 years (95%CI 14.1–14.7) free of mobility limitation at age 60 (Table 3) compared to 11.2 years (95%CI 10.9–11.5) for females of the same age. Females lived significantly fewer years free of limitation than males at all ages (p<0.001), with the gaps ranging from 3.2 years at age 60 to 1.7 years at age 80, and they lived significantly more years with any mobility limitation and with severe limitation (p<0.001). Indeed, females spent twice as many years with any limitation as males did at age 60, 65, and 70 years while the same pattern for severe limitation held for all ages.

**Fig 1:**
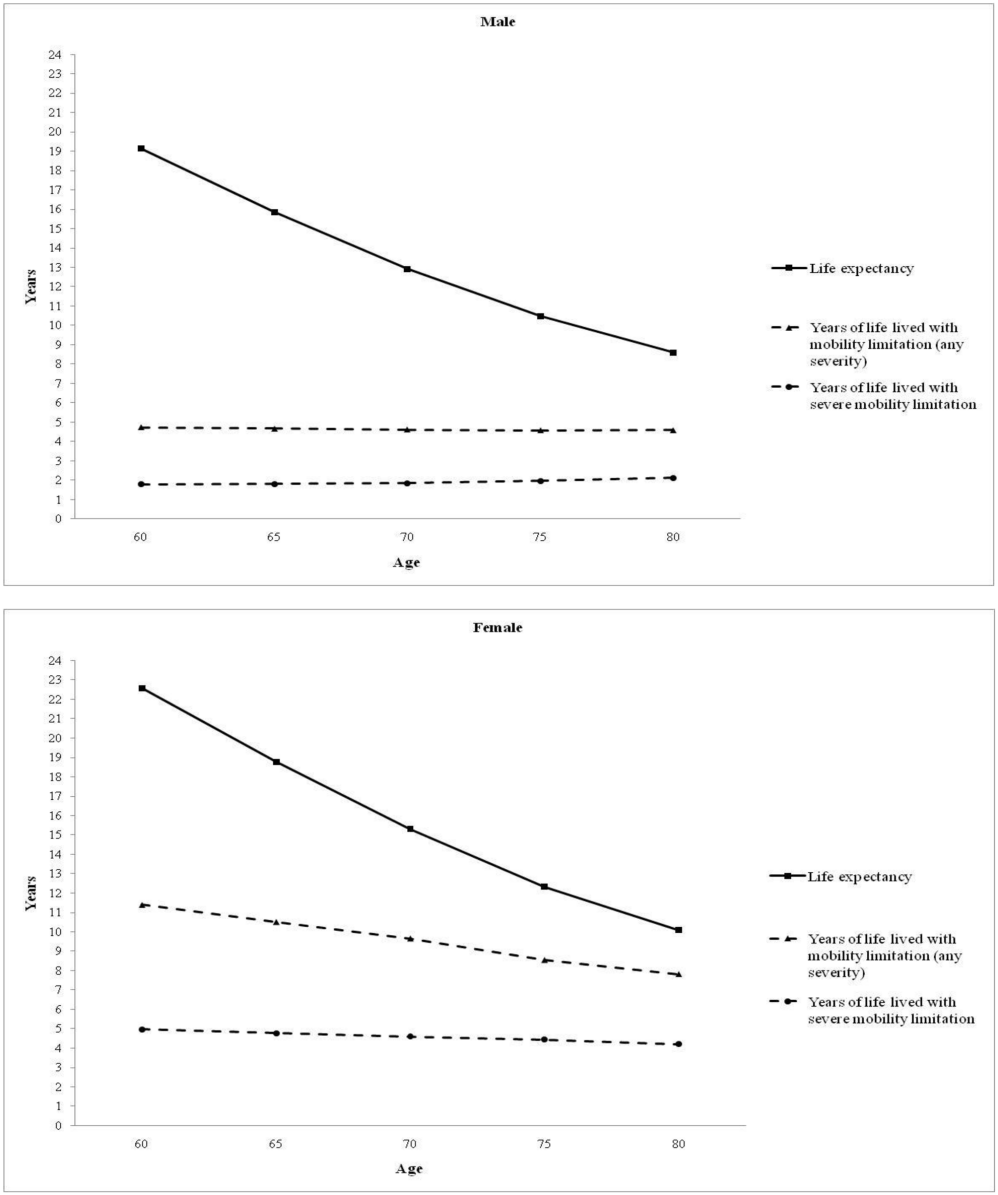
Life expectancy and years of life lived with mobility limitation at age 60, 65, 70, 75, and 80 in Thailand, 2009. Mobility limitation (any severity) was defined as having inability (complete or partial) to do at least one of (i) walking at least 400 meters without resting, and (ii) going up or down a flight of 10 stairs without resting without human assistances or technical aids. Mobility limitation (severe level) was defined as complete inability to do at least one of these two functions, even with assistances or aids.

Fig 1 illustrates life expectancy overall and with mobility limitation at any severity and severe level by age and gender. It was noticeable that years lived with severe limitation were remarkably constant across age at approximately 2 years (males) and 4.5 years (females). However, this pattern for years lived with any limitation was found only in males (at approximately 4.5 years). For females, years of life with any limitation decreased with age at a slower pace than life expectancy.

Life expectancy and years of life in different states of mobility limitation at age 60 by regions, along with the between region rankings, are shown in Table 4 (males) and Table 5 (females). There was a 1.3 year differences in life expectancy at age 60 for males in 2009 between the four regions and Bangkok, from 18.7 years in Bangkok to 20.0 years in the South. For females, the difference was greater (2.7 years), from 21.7 years in the North to 24.6 years in the South. Regional inequalities in years with and without mobility limitations were significantly greater than inequalities in life expectancy. Moreover, significant differences were evident between the regions having the most and the least years lived free of mobility limitation, years lived with any limitation, and years lived with severe limitation. The pattern of regional differences in years lived free of mobility limitation was consistent across genders, with the Central region ranking the highest and the North East ranking the lowest. However, differences in years of life with any mobility limitation varied by gender, with Bangkok ranking the lowest and the North East the highest for males and the Central ranking the lowest and the South the highest for females. For severe limitation, the Central region ranked the lowest for males and the North East ranked the lowest for females, whilst both males and females in the South had the most years lived with severe limitation.

**Table 4:** Life expectancy, number of years lived in different states of mobility limitation (95%CI), and proportion of years lived with mobility limitation to life expectancy at age 60 for Thai males by regions, 2009.

Regions	Life expectancy	Years lived			% of years lived with mobility limitation to life expectancy	
		free of mobility limitation*	with mobility limitation (any severity)**	with severe mobility limitation***	any severity	severe limitation
Bangkok	18.71 [1]	15.45 (14.62–16.27) [4]	3.27 (2.44–4.09) [1]	1.37 (0.72–2.02) [2]	17	7
Central	19.38 [4]	16.06 (15.56–16.55) [5]	3.32 (2.83–3.82) [2]	0.85 (0.57–1.13) [1]	17	4
North	18.82 [2]	13.91 (13.40–14.43) [2]	4.90 (4.39–5.42) [3]	2.18 (1.64–2.48) [4]	26	12
NorthEast	18.90 [3]	12.85 (12.31–13.39) [1]	6.05 (5.51–6.59) [5]	2.06 (1.64–2.48) [3]	32	11
South	19.97 [5]	14.93 (14.36–15.49) [3]	5.05 (4.48–5.62) [4]	2.31 (1.87–2.75) [5]	25	12

[rank between regions, 1 = lowest, 5 = highest]

*significant differences between the North East and the Central (p<0.05),

**significant differences between Bangkok and the North East (p<0.05),

***significant differences between the Central and the South (p<0.05)

**Table 5:** Life expectancy, number of years lived in different states of mobility limitation (95%CI), and proportion of years lived with mobility limitation to life expectancy at age 60 for Thai females by regions, 2009.

Regions	Life expectancy	Years lived			% of years lived with mobility limitation to life expectancy	
		free of mobility limitation*	with mobility limitation (any severity)**	with severe mobility limitation***	any severity	severe limitation
Bangkok	22.87 [3]	12.94 (11.77–14.11) [4]	9.94 (8.77–11.11) [2]	6.10 (4.95–7.25) [4]	43	27
Central	23.10 [4]	13.44 (12.71–14.16) [5]	9.67 (8.94–10.39) [1]	4.37 (3.72–5.02) [2]	42	19
North	21.74 [1]	10.83 (10.19–11.48) [2]	10.91 (10.26–11.55) [3]	5.51 (4.90–6.13) [3]	50	25
NorthEast	21.86 [2]	9.06 (8.40–9.71) [1]	12.81 (12.15–13.46) [4]	4.32 (3.65–5.00) [1]	59	20
South	24.58 [5]	11.75 (11.01–12.49) [3]	12.83 (12.09–13.56) [5]	6.18 (5.47–6.89) [5]	52	25

[rank between regions, 1 = lowest, 5 = highest]

*significant differences between the North East and the Central (p<0.05),

**significant differences between the Central and the South (p<0.05),

***significant differences between the North East and the South (p<0.05)

When comparing years lived with mobility limitation as a proportion to life expectancy by region, the region with the longest life expectancy and most years lived with mobility limitation ranked the highest in males (Table 4) and the second highest in females (Table 5).

## Discussion

This is the first study at a national level in Thailand to examine gender and regional differences in health expectanciesbased on a measure of mobility. We found that females had longer life expectancy but lived fewer years free of mobility limitation and more years with limitation than males at all ages. In both genders, years lived with severe limitation were remarkably constant across age. There were significant regional inequalities in years lived without and with mobility limitation. The pattern of differences in years free of mobility limitations was consistent across genders, with the Central region ranking the best and the North East ranking the worst. Finally, both males and females in the South, with the longest life expectancy, had the most years of life with severe mobility limitation.

Few studies in Asia focus on mobility limitation, but all are methodologically comparable to ours in terms of data and calculation method. In a study in Thailand using data from the 2007 national survey of the elderly, males and females at age 60 years spent 6.7 years (33.1% of life expectancy) and 11.8 years (51.8% of life expectancy) with mobility disability, respectively [19], although the measure of mobility incorporated activities such as taking public transport, these being influenced by environment and role expectation. In contrast, in the 2005 national survey of senior citizen in Singapore males and females at age 65 years spent only 0.8 years (4.7% of life expectancy) and 1.8 years (8.8% of life expectancy) with mobility restriction, respectively, with mobility restriction being measured by a question on ability to physically move around [24]. These differences in assessing mobility function and the potential for environmental influences highlight the difficulty in comparing our findings with those of other studies, both within Thailand and internationally.

Our study examined years of life with severe mobility limitation, which have never been examined previously. The most noticeable finding is that years spent with severe levels of mobility restriction appear relatively constant across old age and similar patterns have been found in a previous study in Thailand [19] which measured mobility by performance in squatting, climbing two or three stairs, carrying weight, and taking public transport. The consistency in these findings might link to the fact that most causes of mobility limitation are usually associated with the ageing process. Thus years with mobility limitation form an increasing proportion of the reducing life expectancy with age. The findings presented here might imply that on average 2 years (for males) and 4.5 years (for females) of mobility aids (such as wheelchairs, mobility scooters, etc.), and appropriate age-friendly infrastructure (such as ramps for wheelchairs, elevator, etc.) are essential for every person aged 60 years and over to remain mobile.The World Health Organization framework of public health actions for Healthy Ageing highlights both the role of the supportive environment in enabling functional ability and the targeting of a life-course period with declining intrinsic capacity [25].

Our findings suggest that the longer lives of older females are not necessarily free of mobility limitation and confirm other studies which have consistently shown that females spend a greater proportion of their life expectancy with disability or mobility restriction. According to Oksuzyan et al. [26], this phenomenon is called the female-male health-survival paradox. Multiple causes have been suggested, including hormonal, autoimmune, and genetic differences between the genders, though gender differences in lifestyle factors and health behaviours might also contribute [26,27]. In addition, gender differences in morbidity may be linked to biases in reporting health status because gender stereotypes and social roles make it culturally more acceptable for females to have and report illness and health problems [26,27].

Apart from gender differences, this study shows inequalities in health expectancy measured by mobility functions between the regions in Thailand and Bangkok. These regional inequalities might be a result of differences in socioeconomic factors, environmental factors (e.g. urbanization), and health behaviors (e.g. drinking and smoking) that have been evident [28] and could influence mobility limitation and life expectancy and therefore mobility limitation-free life expectancy. However, since life tables are not available by these factors, and the number of regions is too small to undertake a meta-regression, our present cross-sectional analysis cannot take these factors into consideration. The findings also suggest that health expectancies had little relationship to life expectancy across regions. Indeed, the regions with the longest life expectancy appeared to be those with the worst health. In addition, greater differences in health expectancy than in life expectancy could suggest that regional inequalities are more distinct with respect to quality rather than quantity of the remaining life-time.

This study has some limitations. The first limitation lies in its use of the Sullivan method which uses the observed prevalence resulting from the past incidence and mortality experience of each cohort in the survey rather than the current incidence rates [22,29]. Thus, it might not be able to reflect current morbidity patterns. However, the Sullivan method is the most often used because it requires only cross-sectional data and period life tables which are widely available [22,29]. Moreover it has been shown to provide unbiased estimates of health expectancy if the transition rates are stable and smooth over time [29]. The second limitation is that health examination surveys may underestimate the prevalence of mobility limitation as people with severe illness or mobility problems (e.g. hemiplegic conditions) may not have been able to participate. Further, regarding the self-report of mobility performance, it is not clear in the survey whether participants were asked to report their actual performance to do these functions or to judge their capacity. Basically, the level of dependence does not correspond to what they think they can do but to what they actually do [30]. Finally, institutionalized people were not included as there are no publicly available data by age and sex. Nevertheless, in 2010 there were approximately 7,000 institutionalized elderly (combining those in nursing homes and prisons), representing less than 0.1 percent of the total older population [31], so this is unlikely to have had a major effect on our estimates.

This study provides the number of years an older Thai person spends on average with mobility limitation which has important policy implications. Effective care programs, e.g. physical therapy, supportive physical environment as well as mobility aids, and sufficient public health resources are required to provide, on average (both genders combined), for 3 years of severe mobility limitation for every person aged 60 years and over during their lifetime. Our findings can also inform on the average time spent with early mobility restriction before developing severe limitation, a period which is potentially significant for interventions to recover function or to stop or slow progression and health professionals working in the field of human mobility, e.g. physical therapists, play important roles in this period. In addition, this study adds knowledge of gender and regional inequalities in health expectancy in the older Thai population based on a measure of mobility limitation. A deeper analysis of reasons for these differences will help the development of policy to reduce such inequalities.
